# Online calculator to predict early mortality in patient with surgically treated recurrent lower-grade glioma

**DOI:** 10.1186/s12885-022-09225-9

**Published:** 2022-01-28

**Authors:** Ruolun Wei, Chao Zhao, Jianguo Li, Fengdong Yang, Yake Xue, Xinting Wei

**Affiliations:** 1 Department of Neurosurgery, The First Affiliated Hospital of Zhengzhou University, Jianshe East Road, Zhengzhou, China; 2 Department of Neurosurgery, Sanbo Brain Hospital, Capital Medical University, Beijing, China; 3 Department of Neurosurgery, West China Hospital, Sichuan University, Chengdu, China

**Keywords:** Lower-grade glioma, Early mortality, Recurrent glioma, Nomogram

## Abstract

**Purpose:**

The aim of this study was to investigate the epidemiological characteristics and associated risk factors of recurrent lower-grade glioma [LGG] (WHO grades II and III) according to the 2016 updated WHO classification paradigm and finally develop a model for predicting early mortality (succumb within a year after reoperation) in recurrent LGG patients.

**Methods:**

Data were obtained from consecutive patients who underwent surgery for primary LGG and reoperation for tumor recurrence. The end point “early mortality” was defined as death within 1 year after the reoperation. Predictive factors, including basic clinical characteristics and laboratory data, were retrospectively collected.

**Results:**

A final nomogram was generated for surgically treated recurrent LGG. Factors that increased the probability of early mortality included older age (*P* = 0.042), D-dimer> 0.187 (*P* = 0.007), RDW > 13.4 (*P* = 0.048), PLR > 100.749 (*P* = 0.014), NLR > 1.815 (*P* = 0.047), 1p19q intact (*P* = 0.019), IDH1-R132H Mutant (P = 0.048), Fib≤2.80 (*P* = 0.018), lack of Stupp concurrent chemoradiotherapy (*P* = 0.041), and an initial symptom of epilepsy (*P* = 0.047). The calibration curve between the prediction from this model and the actual observations showed good agreement.

**Conclusion:** A nomogram that predicts individualized probabilities of early mortality for surgically treated recurrent LGG patients could be a practical clinical tool for counseling patients regarding treatment decisions and optimizing therapeutic approaches. Free online software implementing this nomogram is provided at https://warrenwrl.shinyapps.io/RecurrenceGliomaEarlyM/

**Supplementary Information:**

The online version contains supplementary material available at 10.1186/s12885-022-09225-9.

## Introduction

Gliomas are among the most common adult brain tumors. Unlike cancers originating from other organs, tumors in the central nervous system (CNS) obey a unique histological and grading system [1]. Following the identification of key oncological alterations providing superior prognostication than pathological identification, the understanding of glioma and other central nervous system tumors has evolved and led to the updated 2016 World Health Organization (WHO) Central Nervous System Tumor Classification. The previous grading of “low-grade glioma,” which referred to WHO grade II gliomas, has been renewed and reclassified as “lower-grade glioma” to encompass WHO grade II and III tumors [2]. Recurrence is typically inevitable for most lower-grade glioma (LGG) patients due to the infiltrative nature of the tumor. An LGG that has been resected in a prior surgery may recur in their initial grade or undergo malignant transformation to a higher grade, such as oligodendroglial to anaplastic-oligodendroglial tumors or anaplastic-astrocytoma to secondary glioblastoma (sGBM).

The treatment decision for recurrent LGG has consistently been of concern for neurosurgeons and oncologists. The primary role of reoperation in the management of recurrent low-grade gliomas has not been clarified [3], but studies have confirmed that reoperation provides a significant benefit for patients with recurrent LGG [4]. However, the rationality of reoperation could be questioned if the patient succumbs to death within one year after reoperation of recurrent LGG, especially as salvage chemotherapy may prolong patient post recurrence survival than one year. To this end, in the present study, we generated and validated a nomogram to assist clinical decision-making by distinguishing recurrent LGG patients at high risk of early mortality.

## Material and methods

### Study population

In this retrospective study, adult patients (aged ≥18 years) who underwent primary surgery and histopathology leading to a diagnosis of WHO grade II or III glioma and consecutively underwent reoperation for tumor recurrence between January 2000 and June 2021 at The First Affiliated Hospital of Zhengzhou University were included. All patients included conformed to reoperative and postoperative Karnofsky Performance Status (KPS) scores ≥70. Electronic medical records and, if needed, paper charts with complete data of pre- and postoperative routine tests were used. The exclusion criteria were as follows: 1) a clinical history of chronic diseases more than 1 year; 2) obvious systemic diseases; 3) patients with other malignancies; 4) no history of receiving chemotherapy or radiotherapy before the operation; and 4) perioperative surgery-related mortality. The final cohort included a total of 106 patients. The following variables were obtained from the cohort and included in the study: age at diagnosis, sex, initial symptoms, oncology markers, tumor size, tumor location and laboratory data. The study protocol was approved by the Ethics Committee of the First Affiliated Hospital of Zhengzhou University, and the requirement for signed informed consent from patients was waived due to the retrospective nature of the analysis. All patient data were treated with confidentiality, in accordance with the Declaration of Helsinki.

### Histological evaluation

Histological evaluations were performed on H&E-stained archival slides. All cases were reviewed by neuropathologists according to the latest WHO classification criteria for tumors of the CNS, and the definitive diagnosis was reaffirmed according to the 2016 WHO classification.

### Immunohistochemical staining and analyses

Immunohistochemical staining was performed on an automated immunohistochemical staining system (BenchMark XT, USA. Stained tissue microarray slides were scanned with a Leica Aperio AT2 scanner at 400× magnification. Digital images were analyzed by Leica Aperio ImageScope software with the Nuclear v9 algorithm. The following biomarkers were recorded: 1) IDH1-R132H (isocitrate dehydrogenase 1), 2) 1p19q, 3) P53, 4) ATRX, and 5) Ki67. IDH1 and 1p19q codeletion and alpha thalassemia/mental retardation syndrome X-linked (ATRX) status were scored as positive or negative. P53 status was quantified as the percentage of stained nuclei: less than 10% of stained nuclei indicated an absence of immunoreactivity; 10–30% indicated a score of 1+; 30.1–50% indicated a score of 2+; and more than 50% indicated a score of 3+. Scores of − 1 or 1+ were regarded as P53 negative, and 2+ and 3+ were regarded as P53 positive. The Ki-67 index was also calculated according to the percentage of Ki-67 positive tumor cells present in the sample.

### Laboratory test analyses

Preoperative laboratory markers of the first operation included NLR: neutrophil-to-lymphocyte ratio, PLR: platelet-to-lymphocyte ratio, LMR: lymphocyte-to-monocyte ratio, RDW: red blood cell distribution width, FIB: fibrinogen level, and DD: D-dimer level.

### Follow-up

Patients were followed by clinical and radiological exams periodically. Tumor recurrence or progression was defined using Response Assessment in Neuro-Oncology (RANO) criteria with both clinical status deterioration and radiologic changes on MRI.

Overall survival (OS) was defined as the interval from the date of initial diagnosis (data of first surgery) to the date of death. Time to recurrence (TTR) was defined as the interval between the date of initial diagnosis (data of first surgery) and the progression of disease. Post recurrence survival (PRS) was defined as the survival after recurrence and reoperation. The interval was censored at the last follow-up visit. The patients’ vital status and date of last follow-up were last updated on May 21, 2021.

Survival analyses were performed using the Kaplan–Meier method. Survival distributions were compared using log-rank tests.

### Statistical method

For continuous variables, independent sample t tests were used to compare distributions. For categorical variables, the chi-squared test or Fisher’s exact test was used as appropriate. The optimal cutoff value for each hematological marker was determined by the best area under the curve (AUC) in receiver operating characteristic (ROC) curve analysis. Patients were classified binarily according to the cutoff values. To filter out early mortality-associated factors from all covariates, univariable Cox analysis was performed, and all significant variables from the analysis were included in multivariate Cox analysis. The hazard ratio and *P* value from the univariate and multivariate Cox analyses are demonstrated in a forest plot. The Kaplan–Meier method was used to generate survival curves, and the log-rank test was used for comparison. Based on the multivariate Cox analysis, a model for predicting the probability of early mortality was generated and visualized as a nomogram. The performance of the prediction model was assessed by comparing the nomogram-predicted versus observed Kaplan–Meier estimates of survival probability.

Statistical analyses were performed using SPSS software (version 20.0) and R software (version 3.3.0). The following R packages were used: “rms” (Frank E Harrell), “plyr” (Hadley Wickham), “survival” (Terry M Therneau), “survminer” (Alboukadel Kassambara), “stargazer” (Marek Hlavac), “neuralnet” (Stefan Fritsch), “DynNom” (Amirhossein Jalali), “pROC” (Xavier Robin), “forestplot” (Max Gordon) (Thomas Lumley), and “regplot” (Roger Marshall). In two-sided tests, *P* < 0.05 was considered statistically significant.

## Result

### Patient characteristics

The clinical characteristics of the cohort are shown in Table 1. The average patient age was 40.09 years, with a range from 18 to 70. A total of 69.8% of the patients were male. The tumor locations from most to least frequent were the frontal lobe for 38 (35.9%), temporal lobe for 21 (19.8%), multiple lobes for 28 (26.4%) and other locations, including the parietal lobe, occipital lobe, cerebellum for 19 (17.9%) patients. A total of 52.8% had epilepsy as the initial symptom. The WHO grade at first diagnosis was II for 77 (72.6%) and III for 29 patients (27.4%). The pathology types at first diagnosis were astrocytoma for 62 (58.5%) and oligodendroglioma for 44 patients (41.5%). During follow-up, malignant transformation of the tumor was diagnosed for 33 (31.1%) patients.

**Table 1:** Summary of Clinical Characteristics of LGG Patients Who Undergo Recurrence

Characteristic	All Patients, *N* = 106
**Age, years**	
Average	40.09
Range	18–70
**Gender, n (%)**	
Female	32 (30.2%)
Male	74 (69.8%)
**Location, n (%)**	
Frontal	38 (35.9%)
Temporal	21 (19.8%)
Multi	28 (26.4%)
Other	19 (17.9%)
**Initial symptom, n (%)**	
Epilepsy	56 (52.8%)
Other	50 (47.2%)
**First diagnosed grade, n (%)**	
WHO II	77 (72.6%)
WHO III	29 (27.4%)
**First diagnosed pathology type, n (%)**	
Astrocytoma	62 (58.5%)
Oligodendroglial	44 (41.5%)
**Malignant transform, n (%)**	
Yes	33 (31.1%)
No	73 (68.9%)
**P53, n (%)**	
Mutant	77 (72.6%)
Wide	29 (27.4%)
**1p/19q Codeletion, n (%)**	
Yes	47 (44.3%)
No	59 (55.7%)
**IDH1-R132H, n (%)**	
Mutant	73 (68.9%)
Wide	33 (31.1%)
**ATRX, n (%)**	
Mutant	66 (62.3%)
Wide	40 (37.7%)
**Ki-67, n**	
Average	17.04
Range	0–80
**Adjuvant therapy, n (%)**	
Stupp	68 (64.2%)
Other	38 (35.8%)
**Blood work, average (IQR)**	
NLR,	2.820 (0.667–21.298)
PLR,	128.653 (41.026–578.723)
LMR,	4.191 (0.181–21.667)
RDW, %	13.65 (12.0–23.6)
FIB, g/L	2.74 (1.26–14.30)
DD, mg/L	0.160 (0.005–1.080)
**TTR, days**	
Average	1806.58
Range	177–6310
**PRS, days**	
Average	768.86
Range	39–1981
**OS, days**	
Average	2575.43
Range	263–8176
**Survival Status, n (%)**	
Alive	42 (39.6%)
Dead	64 (60.4%)

*IDH1* Isocitrate dehydrogenase 1, *ATRX* Alpha thalassemia/mental retardation syndrome X-linked, *NLR* the neutrophil-to-lymphocyte ratio, *PLR* the platelet-to-lymphocyte ratio, *LMR* the lymphocyte-to-monocyte ratio, *RDW* Red blood cell distribution width, *FIB* fibrinogen, *DD* D-dimer, *IQR* Interquartile range, *TTR* Time to recurrence, *PRS* Post recurrence survival, *OS* Overall survival

P53 mutation accounted for 77 (72.6%) cases, 1p/19q codeletion accounted for 47 (44.3%) cases, IDH1^R132H^ mutation accounted for 73 (68.9%) cases, ATRX mutation accounted for 66 (62.3%) cases, and the average Ki-67 index was 17.04 with a range from 0 to 80. A total of 64.2% of the patients received Stupp standard radiochemotherapy after the first diagnosis. The average values of the laboratory test markers were as follows: NLR: 2.820 (IQR 0.667–21.298), PLR: 128.653 (IQR 41.026–578.723), LMR: 4.191 (IQR 0.181–21.667), RDW: 13.65 (IQR 12.0–23.6), FIB: 2.74 (IQR 1.26–14.30), DD: 0.160 (IQR 0.005–1.080).

During follow-up, the average OS after the first diagnosis was 2575.43 days (263–8176 days), the average TTR between the first diagnosis and tumor progression was 1806.58 days (177–6310 days), and the average PRS was 768.86 days (39–1981 days).

The cutoff values of the six hematological factors for OS are shown in Supplementary Table 1 (NLR-1.815, PLR-100.749, LMR-3.029, RDW-13.4, FIB-2.80, DD-0.187). According to these values, the patients were classified into binary categories. Table 2 compares the preoperative risk factors for postoperative death within 1 year after LGG recurrence. Twenty-three patients (21.7%) died within 1 year after reoperation. Early mortality was significantly associated with epilepsy as an initial symptom, 1p/19q codeletion, NLR, PLR, RDW, FIB and DD.

**Table 2:** Comparison of Preoperative Risk Factors for Postoperative Death Within 1 Year After Tumor Recurrence

Characteristics	Death Within 1 Year (23)	Death After 1 Year (83)	P value
**Age, years**			
Average	41.83	39.61	0.618
Range	24–68	18–70	
**Gender, n (%)**			
Female	5 (21.7%)	27 (32.5%)	0.233
Male	18 (78.2%)	56 (67.5%)	
**Location, n (%)**			
Frontal	8 (34.8%)	30 (37.3%)	0.953
Temporal	4 (17.4%)	17 (20.5%)	
Multi	6 (26.1%)	22 (26.5%)	
Other	5 (21.7%)	14 (15.7%)	
**Initial symptom, n (%)**			
Epilepsy	17 (73.9%)	39 (47.0%)	0.019
Other	6 (26.1%)	44 (53.0%)	
**First diagnosed grade, n (%)**			
WHO II	16 (69.6%)	61 (73.5%)	0.447
WHO III	7 (30.4%)	22 (26.5%)	
**First diagnosed pathology type, n (%)**			
Astrocytoma	16 (69.6%)	46 (55.4%)	0.164
Oligodendroglial	7 (30.4%)	37 (44.6%)	
**Malignant transform, n (%)**			
Yes	8 (34.8%)	25 (30.1%)	0.425
No	15 (65.2%)	58 (69.9%)	
**P53, n (%)**			
Mutant	19 (82.6%)	58 (69.9%)	0.172
Wide	4 (17.4%)	25 (30.1%)	
**1p/19q Codeletion, n (%)**			
yes	6 (26.1%)	41 (49.4%)	0.038
No	17 (73.9%)	42 (50.6%)	
**IDH1, n (%)**			
Mutant	15 (65.2%)	58 (69.9%)	0.425
Wide	8 (34.8%)	25 (30.1%)	
**ATRX, n (%)**			
Mutant	15 (65.2%)	51 (61.4%)	0.470
Wide	8 (34.8%)	32 (38.6%)	
**Ki-67, n**			
Average	16.13	17.95	0.870
Range	2–80	0–70	
**Adjuvant therapy, n (%)**			
Stupp	10 (43.5%)	58 (69.9%)	0.019
Other	13 (56.5%)	25 (30.1%)	
**NLR, n (%)**			
≤1.815	4 (17.4%)	32 (38.6%)	0.046
>1.815	19 (82.6%)	51 (61.4%)	
**PLR, n (%)**			
≤100.749	4 (17.4%)	36 (43.4%)	0.018
>100.749	19 (82.6%)	47 (56.6%)	
**LMR, n (%)**			
≤3.029	6 (26.1%)	27 (32.5%)	0.375
>3.029	17 (73.9%)	56 (67.5%)	
**RDW, n% (%)**			
≤13.4	11 (47.8%)	59 (71.1%)	0.035
>13.4	12 (52.2%)	24 (28.9%)	
**Fib, g/L, n (%)**			
≤2.80	10 (43.5%)	58 (69.9%)	0.019
>2.80	13 (56.5%)	25 (30.1%)	
**DD, mg/L, n (%)**			
≤0.187	11 (47.8%)	61 (73.5%)	0.020
>0.187	12 (52.2%)	22 (26.5%)	
**TTR, days**			
Average	1777.48	1814.64	0.908
Range	216–5425	177–6310	

*IDH1* isocitrate dehydrogenase 1, *ATRX* alpha thalassemia/mental retardation syndrome X-linked, *NLR* the neutrophil-to-lymphocyte ratio, *PLR* the platelet-to-lymphocyte ratio, *LMR* the lymphocyte-to-monocyte ratio, *RDW* red blood cell distribution width, *FIB* fibrinogen, *DD* D-dimer, *IQR* interquartile range, *TTR* time to recurrence, *OS* overall survival

### Univariate and multivariate analyses

**Fig. 1:**
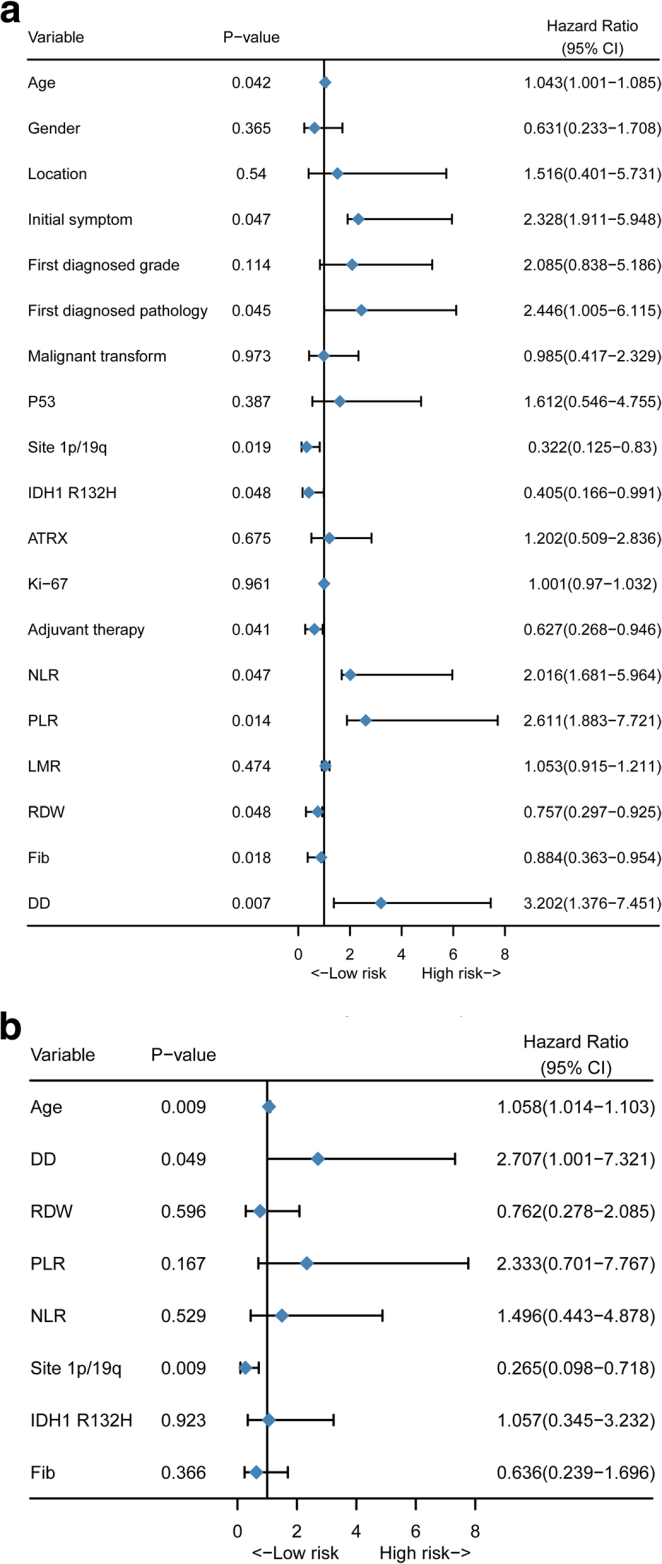
Preoperative predictive factors for 1-year mortality in patients with surgically treated recurrent lower-grade glioma. (**A**) Forest plot of univariate Cox regression analysis of all clinical covariates; (**B**) Forest plot of multivariate Cox regression analysis of significant clinical covariates

The univariate and multivariate Cox regression results are visualized in Fig. 1A and Fig. 1B. Eight predictive variables were associated with a higher risk of early death within 1 year after reoperation in the univariate Cox analysis: older age (*P* = 0.042), higher DD (*P* = 0.007), higher RDW (*P* = 0.048), higher PLR (*P* = 0.014), higher NLR (*P* = 0.047), 1p19q intact (*P* = 0.019), IDH1^R132H^ mutation (P = 0.048), lower FIB (*P* = 0.018), lack of Stupp concurrent chemoradiotherapy (*P* = 0.041), and an initial symptom of epilepsy (P = 0.047). In multivariate Cox analysis, age (HR = 1.058, 95% CI 1.014–1.103; *P* = 0.009), DD (HR = 2.707, 95% CI = 1.001–7.321) and 1p19q (HR = 0.265, 95% CI 0.098–0.718; P = 0.009) remained independently associated with early mortality after reoperation.

### Nomogram and validation

**Fig. 2:**
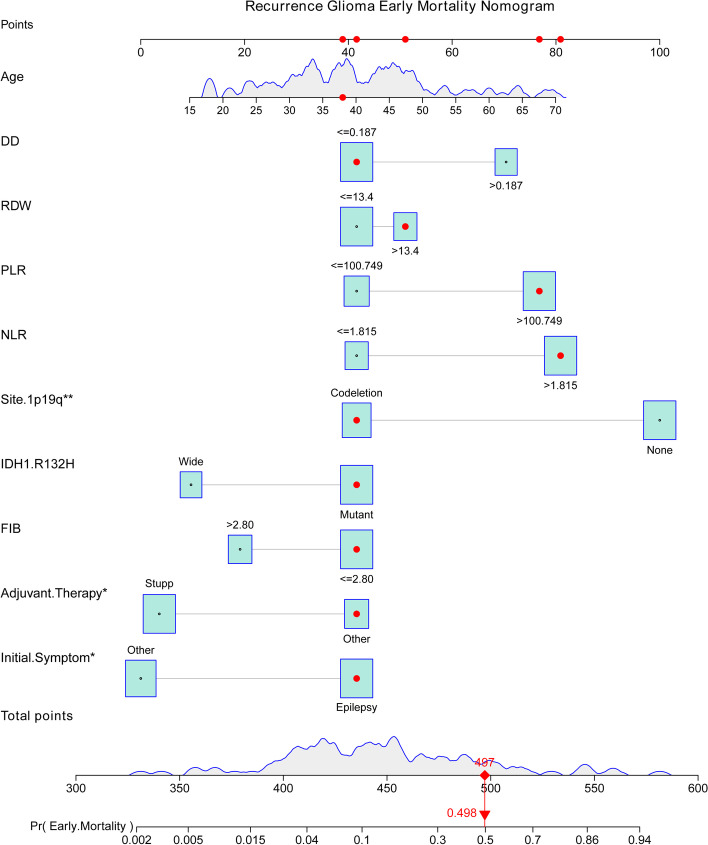
Establishment of an overall survival nomogram for predicting the probability of 1-year mortality in patients with surgically treated recurrent lower-grade glioma

**Fig. 3:**
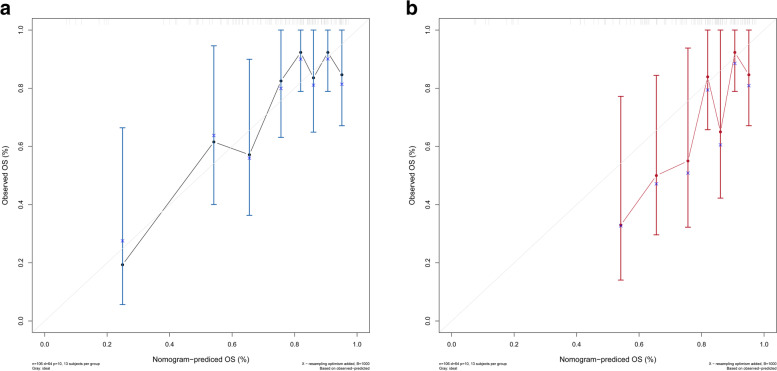
Calibration curves for verifying the prediction accuracy of the nomogram for 5-year survival (**A**) and 7-year survival (**B**)

Estimated probabilities for early mortality for surgically treated recurrent tumors were obtained by the sum of each variable score (Fig. 2). The calibration plot for the probability of survival for 5 years and 7 years after the first diagnosis showed optimal agreement between the prediction from the nomogram and the actual observation (Fig. 3A, Fig. 3B). An online calculator for the final nomogram is available at https://warrenwrl.shinyapps.io/RecurrenceGliomaEarlyM/.

### Survival analysis for independent markers

**Fig. 4:**
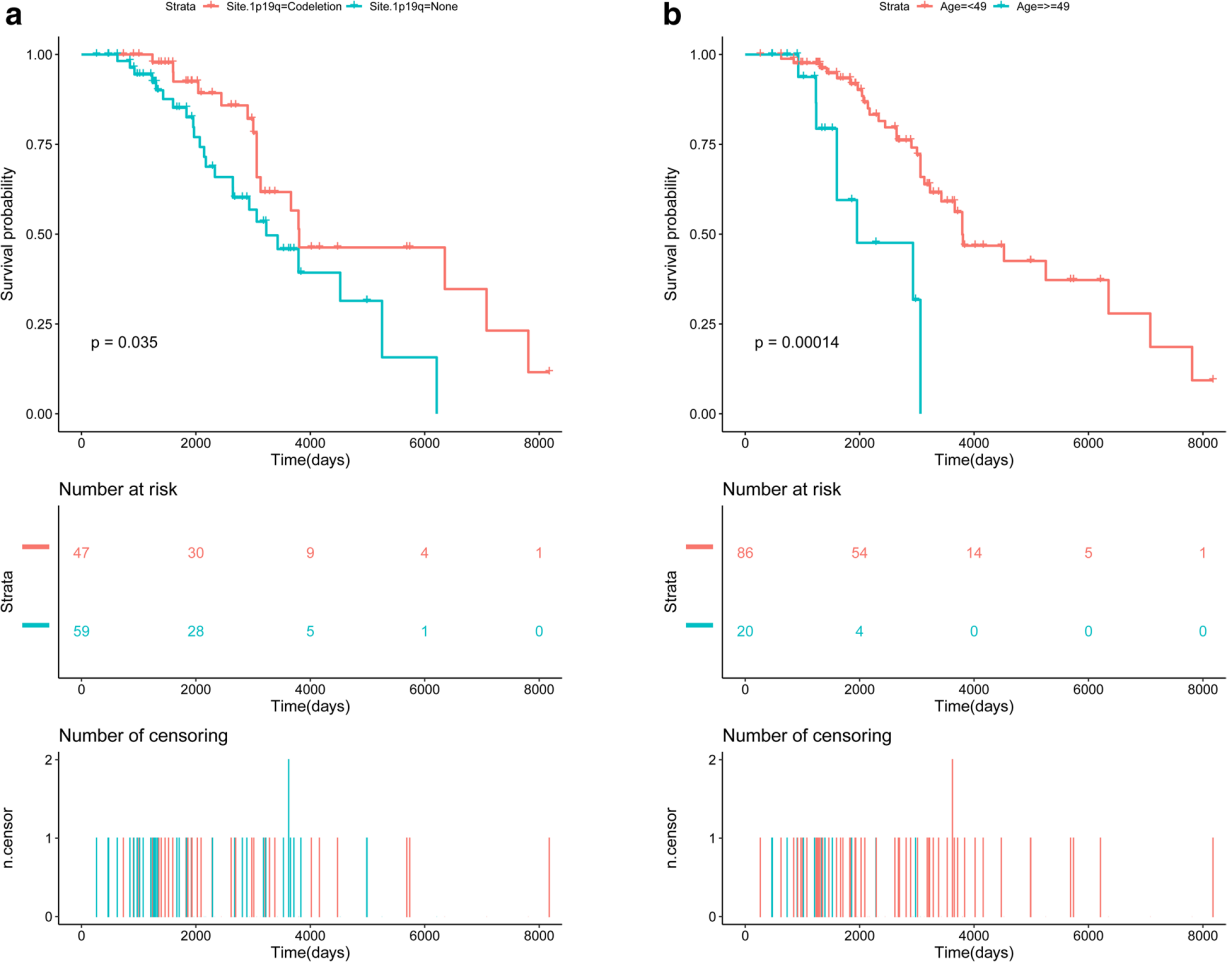
Comparison of survival curves for surgically treated recurrent lower-grade glioma stratified by different variables. (**A**) Kaplan–Meier curves of overall survival for patients in groups stratified by 1p19q codeletion; (**B**) Kaplan–Meier curves of overall survival for patients in groups stratified by age

For the independent associated factors according to multivariate Cox analysis, recurrent LGG patients without 1p19q codeletion showed a significantly poorer prognosis than those with 1p19q codeletion (Fig. 4A), and similarly, patients of older age showed a significantly poorer prognosis than younger patients (Fig. 4B). The results indicate that for recurrent LGG, patient characteristics, preoperative blood test and oncological information of the prior LGG may identify high-risk patients in clinical practice.

## Discussion

Although recent research indicates that LGG patients could gain a survival benefit from repeat surgery after tumor recurrence, surgical treatment has not been widely accepted as a standard recurrent LGG treatment protocol due to an insufficient number of studies. Notably, newly developed salvage radiochemotherapy on late-stage recurrent glioma has demonstrated the potential to prolong OS, expanding the therapy choices for recurrent LGG. Thus, in this study, we provided a tumoral and hematological marker-based nomogram to identify high-risk patients who may succumb to death within a year after reresection of recurrent LGG. Furthermore, this nomogram is implemented in free online software provided for easy use by clinicians worldwide.

During hospital stay, blood tests can contain sufficient significant hematological markers. These hematological markers from peripheral blood tests play a vital role in the prognosis of various malignant tumors [5–7], suggesting that the hemostatic components are interconnected with cancer biology in some ways [8]. In the present study, we included 6 hematological markers with 3 systemic inflammatory markers and 2 coagulation factors from 106 lower-grade glioma patients in this nomogram to assess preoperative risk factors for early death. The DD, RDW, PLR, NLR, and Fib levels were found to be related to early death in recurrent LGG.

As indicators of chronic inflammation, the PLR and NLR show potential to be representative of the characteristics of the tumor microenvironment, and chronic inflammation may foster tumor progression [9]. The NLR is a marker of the systemic inflammatory response and has been found to act as a factor for a poor prognosis in many malignancies, such as colon, bladder, and prostate cancers. In glioblastoma, an NLR > 4 independently leads to a worse prognosis [10]. Further research quantified the prognostic value of PLR, NLR, and LMR based on the IDH mutation status in glioma and noted that a low NLR was associated with a better prognosis in the IDH-wild glioblastoma group, while PLR was predictive of survival in patients with primary glioblastoma and the IDH-wild GBM group [11]. However, the predictive value of the NLR for early death in the LGG cohort has not yet been reported. We believe our results indicate that the preoperative NLR level might represent a cancer-related systemic inflammatory response and may be a factor in predicting early mortality in surgically treated recurrent LGG.

Cancer development and aggressiveness mostly rely on neoangiogenesis and metastasis promoted by cancer cells [12]. Numerous studies have illustrated that coagulation activation is directly related to tumor progression, and mitigating coagulation activation can both prevent hemostatic complications and prolong survival in cancer patients [8]. An abnormal preoperative coagulation state not only indicates a poor general condition but also implies active tumor growth. As a crucial component in hemostasis and fibrinolysis activation, the D-dimer level is often linked with a poor prognosis in several cancers of the body, such as lung, ovarian, gastric and liver cancers [13–15]. Our research indicates that an elevated preoperative plasma value of D-dimer in patients with recurrent LGG is a risk factor for a higher incidence of death within a year after reoperation.

Under the 2016 WHO classification, 1p19q codeletion status is a necessary subtype that must be detected for WHO II-III astrocytoma with an IDH mutation or WHO II-III oligodendroglioma. 1p19q codeletion represents the loss of heterozygosity in the short arm of chromosome 1 (1p) and the long arm of chromosome 19 (19q). The 1p19q state predicts the treatment response to chemotherapy, where codeletion predicted the effectiveness of temozolomide (TMZ) and procarbazine, lomustine, vincristine (PCV) chemotherapy and was associated with a better OS [16]. Recent studies have concluded that 1p19q codeletion has independent significance on the overall survival of LGG patients who experience tumor recurrence and reoperation. A systematic analysis of clinical and biological significances of 1p19q genes indicates in 1p/19q intact tumors, 1p19q-associated risk genes were enriched in processes that promote tumor growth and migration, regulate tumor microenvironment and metabolism and promote drug resistance [17]. We further added the value of the influence of 1p19q in a clinical cohort by demonstrating that patients with an intact 1p19q site may experience earlier-than-expected mortality after reoperation of the recurrent tumor.

Mutations in the isocitrate dehydrogenase (IDH) 1 gene are commonly found in human glioma, with most lower-grade gliomas harboring a recurrent point mutation (IDH1 R132H). After the update of the 2016 WHO classification criteria, the importance of the IDH mutation was highlighted. Harboring the IDH1 mutation is linked to improved survival, and this holds true even in the setting of high-grade gliomas [18]. In this study, a conclusion of an insignificant association between IDH status and early mortality is reached, but we do not believe that this contradicts the findings of large-scale research. With LGG patients’ 5-year survival rates reaching 74% and some patients surviving more than 10 years after active clinical intervention, the survival advantage gained by the IDH mutation is often reflected over a longer period. The setting of 1-year time for early mortality in our study is a better reflection of reoperation-danger factors for the patient than the characteristic differences of the nature of the tumor.

Clinical presentation, which can be expressed as the initial presence of epilepsy, is a strong prognostic factor [19]. Epilepsy is the most common presenting symptom, observed in 80% of LGG patients [20]. We found that 52.8% of our cohort presented with epilepsy as the first symptom, which is in accordance with the findings with the TCGA dataset (53.2%) and the OBTS dataset (61.2%) [21]. A large cohort study reported that the presence of seizures at diagnosis was associated with more favorable outcomes, which might be due to the younger age of those whose initial symptoms was epilepsy. This appears contrary to our result, but the same study noted that a history of epileptic seizures at diagnosis was an independent predictor for malignant transformation: malignant-free survival was approximately 65 months and 40 months in LGG patients with and without a history of epileptic seizures at diagnosis, respectively [22]. Considering that a selective bias of inclusion of full recurrent LGG with an appreciable amount of malignant transformed glioma in our cohort, we believe that our result is robust.

One year mortality in reoperation of LGG patients is associated with various factors. Part of patients may succumb in a short while after the reresection. In view of our study result, we consider the adverse factors such as the abnormal systemic inflammatory and coagulation factors are reflecting the inferior state of the internal environment. The perioperative management and evaluation of glioma patients should balance the tumor characteristics and patients, in other words, treat the disease and the patients as while. Therefore, preoperative evaluation of patients’ internal environment status and selecting appropriate therapy plan such as radical reoperation or salvage chemotherapy may contributes to improve oncologic outcome.

In all patients from our institution who underwent surgical resection of the primary tumor, a portion of patients were treated with a standard Stupp concurrent chemoradiotherapy plan, while others received TMZ, radiotherapy monotherapy, or close observation. The formulation of the adjuvant treatment plan is based on up-to-date glioma treatment guidance, and our study only illustrated the prediction of early mortality in patients with surgically treated, recurrent LGG based on these related factors. The enrolled patients in this study were identified at a single institution, and the prediction model may not be applicable to a multicenter cohort. As such, the result of the online calculator cannot be interpreted, as a nonsurgery plan should be preferred or used as a basis to change the treatment strategy for recurrent diffuse LGGs. Additionally, as we were limited by the wide time span of the longitudinal follow-up, the sample size was not sufficiently large, which may have led to bias in selection and analysis. Furthermore, due to funding and tissue limits, the study was limited to as a single-center cohort. Therefore, we disclosed all the initial data and original code, hoping to contribute to build a more precise and universal prediction model with a larger cohort and multicenter data.

## Conclusion

In summary, we developed a nomogram to enable personalized estimation of 1-year mortality for patients with surgically treated recurrent lower-grade glioma based on patient clinical information, hematological markers and oncological factors. To facilitate the clinical use of this nomogram, free online software for its implementation is provided (https://warrenwrl.shinyapps.io/RecurrenceGliomaEarlyM/).

## Supplementary Information

**Additional file 1 Supplementary Table S1.** The optimal cutoff values of hematological factors for overall survival by Receiver operating characteristic (ROC) curve analysis.

## Data Availability

The data that support the findings and the R-studio code that was generated in this study are openly available in Zenodo (DOI:10.5281/zenodo.5558404).

## Abbreviations

LGG: Lower-grade glioma
WHO: World Health Organization
CNS: Central Nervous System
sGBM: Secondary Glioblastoma
KPS: Karnofsky Performance Status
IDH1: Isocitrate dehydrogenase 1
ATRX: Alpha thalassemia/mental retardation syndrome X-linked
NLR: Neutrophil-to-Lymphocyte Ratio
PLR: Platelet-to-Lymphocyte Ratio
LMR: Lymphocyte-to-Monocyte Ratio
RDW: Red blood cell Distribution Width
FIB: Fibrinogen Level
DD: D-Dimer Level
OS: Overall Survival
PFS: Progression-Free Survival
RANO: Response Assessment in Neuro-Oncology
ROC: Receiver Operating Characteristic
AUC: Area Under the Curve
TMZ: Temozolomide
PCV: Procarbazine, lomustine, vincristine
TCGA: cancer genome atlas
OBTS: Ohio Brain Tumor Study
