# Supplementary material for: Online calculator to predict early mortality in patient with surgically treated recurrent lower-grade glioma
Source: BMC Cancer. 2022 Jan 28;22:114. doi: 10.1186/s12885-022-09225-9 (PMC8796632; doi:10.1186/s12885-022-09225-9)
Supplement: Supplementary file 1 — Additional file 1 Supplementary Table S1. The optimal cutoff values of hematological factors for overall survival by Receiver operating characteristic (ROC) curve analysis. [file 12885_2022_9225_MOESM1_ESM.docx]

**Supplementary Table S1.** The optimal cutoff values of hematological factors for overall survival by Receiver operating characteristic (ROC) curve analysis.

| **covariates** | **cutoff values** |
| --- | --- |
| NLR | 1.815 |
| PLR | 100.749 |
| LMR | 3.029 |
| RDW | 13.4 |
| FIB | 2.80 |
| DD | 0.187 |

**Abbreviations:** NLR, the neutrophil-to-lymphocyte ratio; PLR, the platelet-to-lymphocyte ratio; LMR, the lymphocyte -to- monocyte ratio; RDW, red blood cell distribution width; FIB, fibrinogen; DD, D-dimer.
